# Translating clinical notes into quantitative measures—a real-world observation on the response to cholinesterase inhibitors or selective serotonin reuptake inhibitors prescribed to outpatients with dementia using electronic medical records

**DOI:** 10.3389/fphar.2023.1177026

**Published:** 2023-05-10

**Authors:** Jill S. Chotiyanonta, Kengo Onda, Milap A. Nowrangi, Xin Li, Xin Xu, Roy Adams, Constantine G. Lyketsos, Peter Zandi, Kenichi Oishi

**Affiliations:** ^1^ Department of Radiology, Johns Hopkins University School of Medicine, Baltimore, MD, United States; ^2^ Department of Psychiatry and Behavioral Sciences, Johns Hopkins University School of Medicine, Baltimore, MD, United States; ^3^ Richman Family Precision Medicine Center of Excellence in Alzheimer’s Disease, Johns Hopkins University School of Medicine, Baltimore, MD, United States

**Keywords:** dementia, cognitive impairment (dementia), psychiatric symptom, electronic medical record, clinical notes, treatment response, selective serotonin reuptake inhibitor, cholinesterase inhibitor

## Abstract

**Objective:** Cholinesterase inhibitors (CEIs) are prescribed for dementia to maintain or improve memory. Selective serotonin reuptake inhibitors (SSRIs) are also prescribed to manage psychiatric symptoms seen in dementia. What proportion of outpatients actually responds to these drugs is still unclear. Our objective was to investigate the responder rates of these medications in an outpatient setting using the electronic medical record (EMR).

**Methods:** We used the Johns Hopkins EMR system to identify patients with dementia who were prescribed a CEI or SSRI for the first time between 2010 and 2021. Treatment effects were assessed through routinely documented clinical notes and free-text entries in which healthcare providers record clinical findings and impressions of patients. Responses were scored using a three-point Likert scale named the NOte-based evaluation method for Treatment Efficacy (NOTE) in addition to the Clinician’s Interview-Based Impression of Change Plus caregiver input (CIBIC-plus), a seven-point Likert scale used in clinical trials. To validate NOTE, the relationships between NOTE and CIBIC-plus and between NOTE and change in MMSE (Mini-Mental State Examination) before and after medication were examined. Inter-rater reliability was evaluated using Krippendorff’s alpha. The responder rates were calculated.

**Results:** NOTE showed excellent inter-rater reliability and correlated well with CIBIC-plus and changes in MMSEs. Out of 115 CEI cases, 27.0% reported improvement and 34.8% reported stable symptoms in cognition; out of 225 SSRI cases, 69.3% reported an improvement in neuropsychiatric symptoms.

**Conclusion:** NOTE showed high validity in measuring the pharmacotherapy effects based on unstructured clinical entries. Although our real-world observation included various types of dementia, the results were remarkably similar to what was reported in controlled clinical trials of Alzheimer’s disease and its related neuropsychiatric symptoms.

## Introduction

Different types of dementia, including Alzheimer’s disease (AD), are progressive neurodegenerative conditions that impair cognitive function, commonly present with neuropsychiatric symptoms (Lyketsos and Lee, 2004) and eventually require individuals to be under constant care and supervision. Currently, available medications that are commonly used to treat dementia symptoms include cholinesterase inhibitors (CEIs) to address cognitive symptoms and selective serotonin reuptake inhibitors (SSRIs) to address neuropsychiatric symptoms. While SSRIs used for the treatment of depression still remains controversial (Banerjee et al., 2011; Wilkins and Forester, 2016), both CEIs and SSRIs have been shown to have modest but important clinical benefits in the symptomatic treatment of dementia that includes stabilizing agitations and cognitive declines (Seitz et al., 2011; Porsteinsson et al., 2014b; Birks et al., 2015; Birks and Harvey, 2018). However, patients presenting with cognitive and behavioral dementia symptoms respond differentially to these medications. Medication effectiveness varies among individuals (Eagger and Harvey, 1995; Trivedi et al., 2006) in part because patients with cognitive decline can be highly heterogeneous in their underlying pathology (Bullock et al., 2006; Lam et al., 2013).

Although the pharmacotherapeutic effects of anti-dementia drugs and antidepressants used to treat dementia symptoms have been studied under strict inclusion and exclusion criteria in clinical trials, the proportion of outpatients who actually respond to these drugs is still under investigation. Recently published research studies involving a large-scale, retrospective clinical record study examined the effectiveness of CEIs and memantine by collecting cognitive assessment scores of dementia patients from routinely collected clinical data (Vaci et al., 2021). Cognitive assessment scores including Mini-Mental State Examination (MMSE) and Montreal Cognitive Assessment (MoCA) are useful reference standards to track declining cognition in dementia patients. However, these cognitive scores are not assessed at every outpatient visit, and cognitive scores before and after drug use are often unavailable. Selecting eligible patients only from those for whom the records of cognitive scores are available may introduce a selection bias. Moreover, the symptomatic treatment of dementia involves not only the cognitive symptoms but also problematic psychiatric symptoms that are commonly present in dementia patients. Symptoms such as apathy, excessive agitation, and nighttime disruptions affect the quality of life of patients and families. The overall treatment effectiveness of medications addressing problematic cognitive and psychiatric symptoms of dementia patients needs to be examined.

The study aimed to find the relative responder rates of CEIs and SSRIs in an outpatient setting using clinical notes documented by clinicians. To this end, our study focused on utilizing electronic medical records (EMRs) that contain a massive amount of routinely collected clinical data of outpatients with dementia. Though clinical notes are rich with information, extracting medication responses from EMRs remains a challenge. As part of this study, we developed a novel method for annotating and quantifying clinical notes with evidence of response to medication and assessed the validity and reliability of this method. Retrospective analysis of clinical notes in EMRs using this method adds the real-world perspective of pharmacotherapeutic effects on patients with dementia due to various pathologies.

## Methods

### Data source and cohort screening

A retrospective analysis of Johns Hopkins EMRs was performed on patients with dementia who were prescribed CEIs or SSRIs for the first time between 2010 and 2021. Patients of interest were identified from the Richman Family Precision Medicine Center of Excellence in the Alzheimer’s Disease database through the Precision Medicine Analytics Platform, which consists of clinical records of patients seen at the Johns Hopkins Medical Institutes. Clinical providers at this center include behavioral neurologists, neuropsychiatrists, and geriatricians. Because these data are part of a larger dataset used for brain imaging analysis, the inclusion criteria included the following: 1) first documented order of CEIs and/or SSRIs as an index date, 2) aged 50 or older at the time of the index date, 3) had any encounter diagnosis of a cognitive disorder, 4) had brain MRI scans within a year before and after the index date, and 5) had at least one encounter within a year before and after the index date. The diagnosis codes, or ICD-10, of interest, included F01 (vascular dementia (VD)), F02 (dementia in other diseases classified elsewhere), F03 (unspecified dementia), G30 (AD), and G31 (other degenerative diseases of the nervous system). Additionally, the following CEIs that are unrelated to dementia treatment were excluded: neostigmine (Bloxiverz) and pyridostigmine (Mestinon).

### Manual review of EMRs

After the initial screening, patients’ clinical records were manually reviewed in descending order of patient ID, and records whose review was completed by April 2022 were included in this article. Because the screened index dates (i.e., the first prescription-ordered date) did not always match the actual medication start date reported by patients and caregivers, the following cases were excluded: individuals who 1) had started SSRIs/CEIs more than 6 months prior to or after 3 months from the index date, 2) were on serotonin–norepinephrine reuptake inhibitors (SNRIs) less than 3 months before starting SSRIs, 3) had unknown medication start dates, 4) had inaccessible, protected records, 5) had no evaluable follow-ups, 6) reported that they never started the medication, and 7) had medication discontinued due to a change in the original diagnosis.

For those who were not excluded, the available medical records with mentions of SSRIs/CEIs throughout their clinical history were reviewed, and any relevant information in a sentence or paragraph within the clinical notes was collected into a spreadsheet as raw data. A manual review was performed by a research data analyst (JC) who has been trained under a neurologist (KIO).

Certain rules have been established when analyzing clinical notes. Due to the nature of dementia, which involves loss of insight and progressive worsening in cognitive function, clinician comments were prioritized over caregiver comments and caregiver comments were prioritized over patient comments, as available. The medication was marked as effective whenever improvement was noted, even if a decline in the overall condition of the patient was reported later. Moreover, when comments implied both improved and worse and no change at the same time, we prioritized improved or worse over no change (e.g., in comments like “memory slightly worse or around the same”, “slightly worse” were prioritized over “around the same”). Furthermore, for patients who were prescribed more than one SSRI or CEI in their clinical history, we set a washout period of 3 months to account for the possible carryover effects between different medications. Medications used from the same class were considered two separate SSRIs or CEIs only if the switch was made after a 3-month period. This 3-month washout period was also applied whenever a period of medication disuse was reported.

In addition to abstracting clinical comments, age at the start of the medication period, sex, medication start and end dates, dates of treatment effects reported, any adverse event (AE), and the corresponding date were recorded. AEs included any side effects or allergic reactions that occurred during treatment that may or may not be related to medication usage; not available (N/A) was marked when there was no recording of either AEs or medication tolerance. The results and dates of MMSE and MoCA were also recorded whenever possible.

### Quantifying treatment responses

To translate evidence from a clinical note into a simple, quantitative measure of medication response, we used Likert scales. We used a three-point Likert scale named *NOte-based evaluation method for Treatment Efficacy* (NOTE), with the following categories: 1 = improved, 2 = no change, and 3 = worse. For comparison, we also applied a widely used global assessment of anti-dementia drugs called the Clinician’s Interview-Based Impression of Change Plus caregiver input (CIBIC-plus) (Joffres et al., 2000; Stanley et al., 2021). CIBIC-plus consists of a seven-point Likert scale to rate the degree of change after medication usage, which is as follows: 1 = markedly improved, 2 = moderately improved, 3 = slightly improved, 4 = unchanged, 5 = slightly worse, 6 = moderately worse, and 7 = markedly worse. In essence, CIBIC-plus allowed a detailed evaluation of the change in the patient’s condition, while NOTE allowed a simplified evaluation.

We used two versions of these two Likert scales by classifying clinical comments into psychiatric and cognitive notes. Cognitive NOTE and cognitive CIBIC-plus rated a change in cognitive symptoms, and psychiatric NOTE and psychiatric CIBIC-plus rated a change in psychiatric symptoms. Comments like “anxiety better” were classified into psychiatric measures and comments like “memory worse” were classified into cognitive measures. When comments were not readily classifiable to either psychiatric or cognitive, such as “sertraline working well” or “donepezil helpful,” then the psychiatric measure was prioritized for SSRIs, and the cognitive measure was prioritized for CEIs. Of note, because a continued decline in cognition over time is expected, the word “stable” was evaluated differently for psychiatric versus cognitive symptoms. Cognitive comments like “dementia stable” were classified as no change, while psychiatric comments like “agitation/irritation now stable” were classified as improved. Patients using CEIs and SSRIs at the same time were evaluated and scored separately using the available cognitive and psychiatric comments. For both scales, when it was not possible to score a treatment effect, 0 = N/A was marked to indicate not available or applicable. Table 1 summarizes the keywords searched and words found within clinical entries in EMRs that were classified for Likert scales.

**TABLE 1:** Keywords found within EMRs.

Implication	Clinical note keywords
SSRI^a^	SSRIs, citalopram, Celexa, escitalopram, Lexapro, fluoxetine, Prozac, Sarafem, Symbyax, fluvoxamine, Luvox, paroxetine, Paxil, Pexeva, sertraline, Zoloft, vilazodone, and Viibryd
CEI^a^	Cholinesterase inhibitor, donepezil, Aricept, rivastigmine, Exelon, galantamine, Razadyne, and Namzaric
Cognitive assessments^a^	MMSE, Mini-Mental State Exam and MoCA, Montreal Cognitive Assessment
Psychiatric note^b^	Depression, depressed, anxious, anxiety, mood, ruminating thoughts, nighttime disruption, hallucination, focus, attention, concentration, alert, irritation, irritability, irritated, agitation, agitated, behavior, challenging behavior, panic attack, frustration, apathy, communicative, altered, confusion, mood swings, emotional, emotionally, tearful, crying, anger, positivity, sleep, sad, no energy, personality, paranoia, feeling, impulsivity, active, engaged, motivated, hopeful, interactive, interacting, verbal, aware, delusions, aggression, dysphoria, elation, euphoria, indifference, disinhibition, disinhibited, labile, lability, motor disturbances, nighttime behavior, psychiatric, and psychiatric symptoms
Cognitive note^b^	Recall, memory, short-term memory, long-term memory, episodic memory, cognition, cognitive, cognitive performance, cognitive function, cognitive symptoms, dementia, Alzheimer’s, word finding difficulty, sharp, sharper, clarity, memory loss, memory lapses, remembering, repeating him/herself, repeating questions, repetitions, mentation
Improve^c^	Improve, improvement, improved, improving, better, well, doing well, [medication] working well, help, helped, helpful, benefits, less/decreased/improvement in/reduction in [problematic symptoms], [psychiatric symptoms] stable/controlled/under control, remembering more things, no longer depressed, not depressed, does not feel depressed, sharper, more [positive behavior such as hopeful/active/engaging/interactive/verbal], close to normal, almost all the way back to baseline, calmed down, calmer, positive effect, and positive turn around
No change^c^	No change, unchanged, no major/significant change, no benefit, uncertain benefit, no improvement, not noticed a/any difference, not made noticeable difference, [cognitive symptoms] stable/stabilized, about the same, same, not helpful, did not help, didn’t help, ineffective, lack of effect, no significant deterioration, no worsening of symptoms, continued [previously mentioned symptoms], remain, still have [symptoms], has not done much, and [cognitive symptoms] not progressing
Worsen^c^	Worsen, worsened, worse, worst, worsening, more/increased/increasingly/worsening of/advancement in [psychiatric/cognitive symptoms], more trouble, continue to decline, and decline in memory/cognition/focus/concentration
Markedly^d^	Markedly, marked, significant, significantly, much, very, very much, really, great, greatly, quite, dramatic, dramatically, clear, clearly, notable, notably, and noticeably
Moderately^d^	Moderately and moderate
Slightly^d^	Slightly, slight, minimally, minimal, bit, little, little bit, some, and somewhat

^a^ SSRIs, CEIs, and cognitive assessments: various prescription names and cognitive assessments searched within EMRs.

^b^ Psychiatric and cognitive notes: classified comments that were found within clinical notes.

^c^ Improve, no change, or worsen: clinical comments classified for a three-point scale of the NOTE score, which is as follows: 1 = improve, 2 = no change, and 3 = worsen.

^d^ Markedly, moderately, and slightly: clinical comments classified for a seven-point scale, which is as follows: 1 = markedly improved, 2 = moderately improved, 3 = slightly improved, 4 = unchanged, 5 = slightly worse, 6 = moderately worse, and 7 = markedly worse. Keywords for improved, unchanged, and worse are the same as the NOTE classification.

Although multiple dates of similar treatment effects commented (i.e., classified in the same category as improved, no change, or worse) were documented for each case whenever available, the dates of reported cognitive comments that matched the dates of MMSE change were first prioritized; then, the comments that were reported closest to 6 months were prioritized for all other psychiatric and cognitive effects. A 6-month duration was selected to ascertain the effectiveness of CEIs and SSRIs, accounting for the incremental nature of their therapeutic impact, the requirement of several weeks to months for appropriate dosage titration, and the capacity to evaluate the sustainability of the medications’ effects within this time frame.

### Definition of responder, non-responder, and intolerance

SSRI responders were defined as psychiatric cases evaluated as improved. SSRI non-responders were defined as psychiatric cases evaluated as no change or worse. CEI responders were defined as cognitive cases evaluated as improved or stable (i.e., no change). CEI non-responders were defined as cognitive cases evaluated as worse. Intolerance was marked when clinical notes stated that individuals discontinued medication use due to AE(s) or the medication was added to the allergy list. These were marked with a score of 0.5 = IT for both scales to distinguish from N/A cases because intolerance cases naturally lacked evaluable treatment effects.

### Statistical analyses

Inter-rater reliability of the Likert scales was evaluated by having another reviewer (KIO) collect and evaluate information on 30 randomly selected patients from EMRs. For the reliability tests, analyses of the intraclass correlation coefficient (ICC) and Krippendorff’s alpha were performed on psychiatric and cognitive Likert scales of two raters using the psych (version 2.1.9) (Revelle, 2021) and irr (version 0.84.1) (Gamer et al., 2019) libraries in R 4.2.0 (R Core Team, 2022), respectively. ICCs of a two-way random-effects model with a significance set at *p* < 0.05 was calculated based on the ordinal assumption (worsen < no change < improved) by excluding N/A values. Krippendorff’s alpha-reliability was calculated to include missing data (Krippendorff, 2011). For both reliability tests, 95% confidence intervals (CIs) were calculated based on bootstrapping with 500 resampling iterations and the bias-corrected and accelerated (BCa) method, using the boot (version 1.3–28.1) (Davison and Hinkley, 1997; Canty and Ripley, 2022) library. Additionally, Dice coefficients for each NOTE were evaluated.

To determine whether the NOTE introduced in this study correlated well with the established CIBIC-plus scores, the Spearman’s rank correlation test with the significance set at *p* < 0.05 was conducted on responses that were evaluable by both scales.

All MoCA scores were converted to MMSE scores using the MMSE–MoCA conversion table by Roalf et al. (2013) and treated as MMSE scores. The MMSE change was calculated as the difference between a) the secondary MMSE score within 2 weeks of the date when a cognitive clinical comment was made, and b) the initial MMSE score within 2 weeks of drug initiation. The Spearman’s rank correlation test with the significance set at *p* < 0.05 was also performed between NOTE and changes in MMSE from pre- to post-medication use.

For all correlation tests, non-parametric bootstrapping with 10,000 resampling iterations and the BCa method were also performed to estimate 95% CIs using the SciPy (version 1.10.1) (Virtanen et al., 2020) library in Python 3.8.

The proportion of responders, non-responders, and intolerances was analyzed in relation to age, sex, and ICD-10 code. To see if the drug responses were affected by these characteristics, multinomial baseline-category logit model analyses were performed using the VGAM (version 1.1–5) (Yee, 2021) library in R 4.2.0.

## Results

### Demographics and clinical characteristics

**FIGURE 1:**
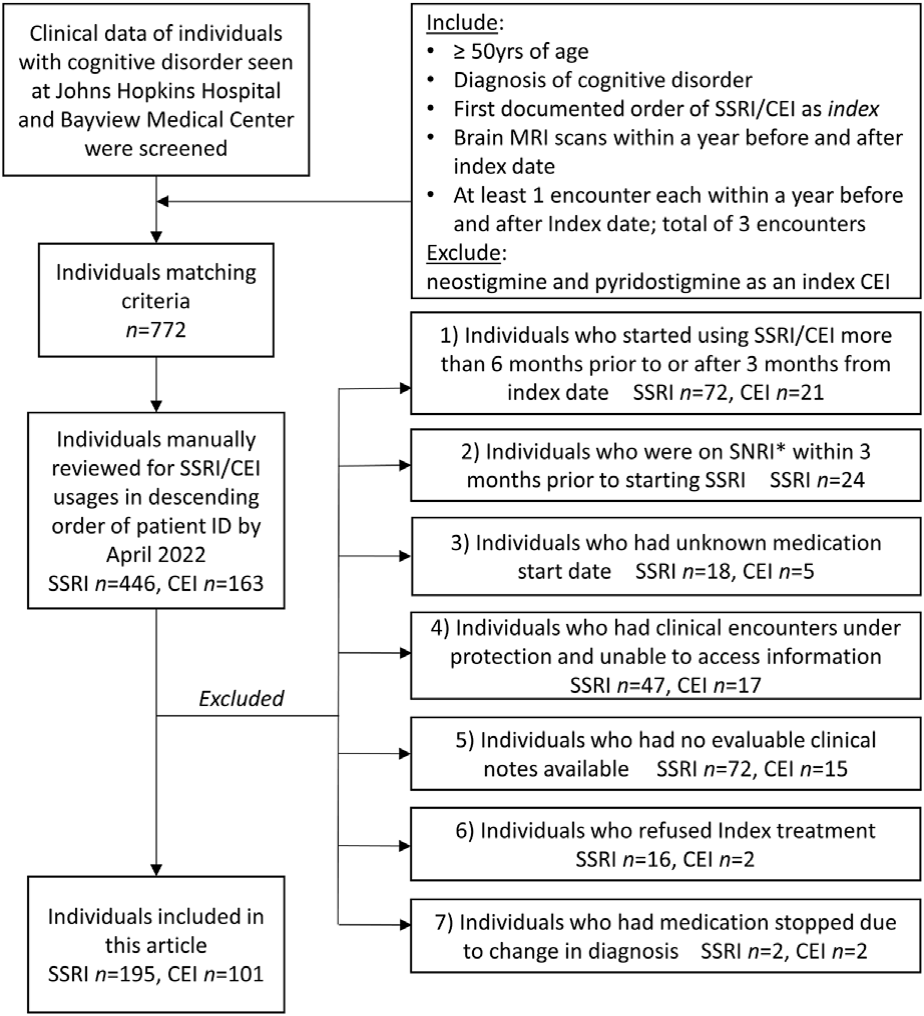
Inclusion and exclusion criteria of patients and each *n* count. *Index* SSRI/CEI and *index date* represent the first identified prescription order and its date, respectively. The excluded individuals and reasons are listed. *SNRI, serotonin–norepinephrine reuptake inhibitor.

Initial screening identified a total of 772 patients of interest. Individuals manually reviewed for clinical notes by April 2022 included *n* = 446 for SSRIs and *n* = 163 for CEIs. Figure 1 summarizes the inclusion/exclusion criteria and the number of patients for each screening stage. After excluding *n* = 251 SSRI and *n* = 62 CEI users due to their medication usage and record unavailability, 195 patients on SSRIs and 101 patients on CEIs were included in this study. Among these individuals, 24 patients used more than one SSRI, and 10 patients used more than one CEI in their clinical history or had a period of disuse between the same classes of medication that goes beyond the 3-month washout period. Considering these cases, a total of 225 SSRI and 115 CEI cases of medication usage were collected from EMRs. Each medication usage was considered an independent observation.

**FIGURE 2:**
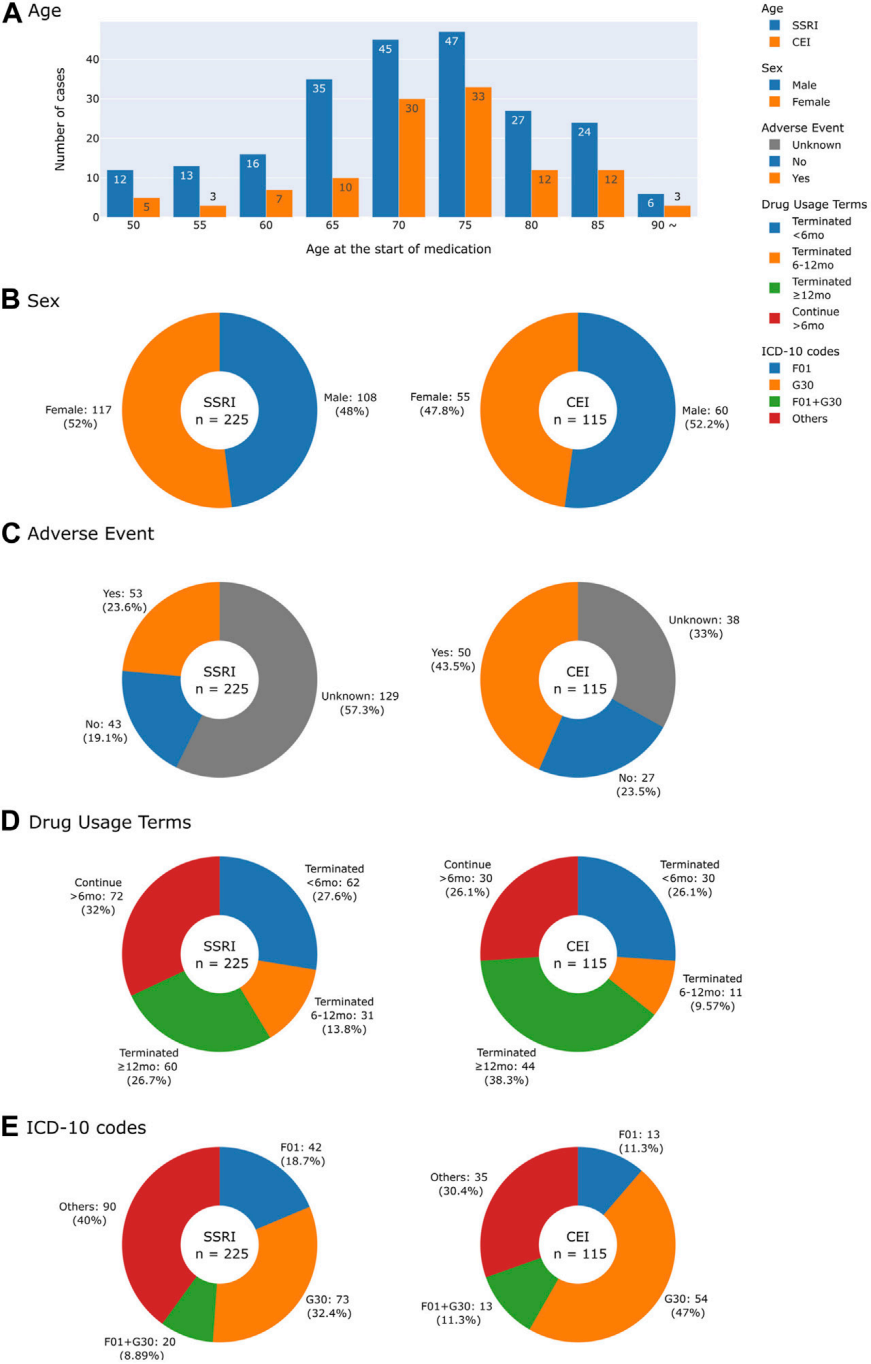
Histogram and pie charts summarizing demographics and clinical characteristics of patients by drug usage. **(A)** Age at the start of medication. **(B)** Sex. **(C)** Adverse events reported. **(D)** Drug usage terms separated into four groups: terminated drug use in less than 6 months, within 6–12 months, after 12 months, and continuing for more than 6 months for those who did not have a record of terminating drug use. **(E)** Collected ICD-10 codes were classified into F01 (vascular dementia), G30 (Alzheimer’s disease), F01 + G30 for vascular and Alzheimer’s mixed dementia, and others for patients with other ICD-10 codes that include F02 (dementia in other diseases classified elsewhere), F03 (unspecified dementia), and G31 (other degenerative diseases of the nervous system).

**FIGURE 3:**
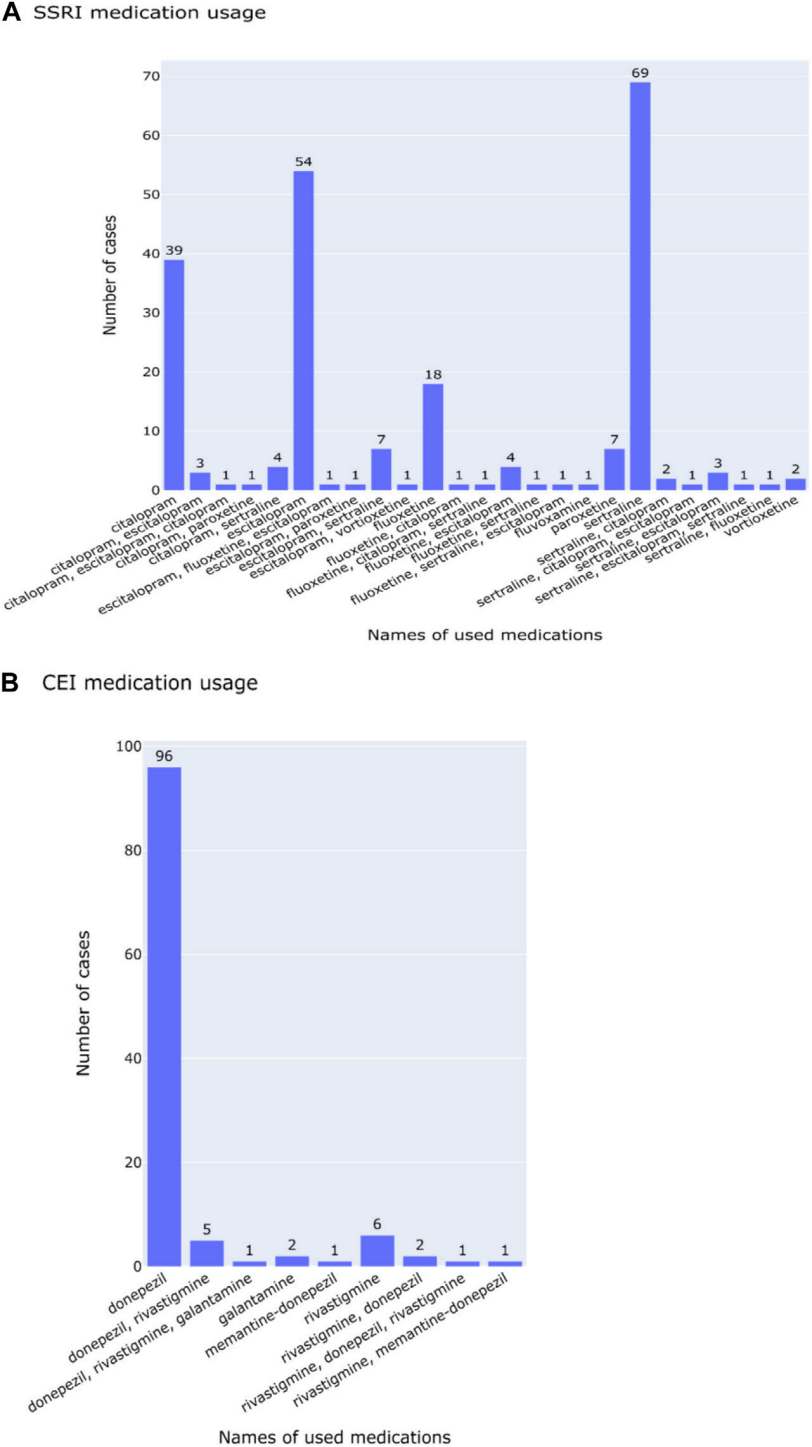
Lists of medications used for **(A)** SSRI and **(B)** CEI cases. Medication names that have more than two drugs are switched within the 3-month washout period and separated by commas in the order of usage.

**FIGURE 4:**
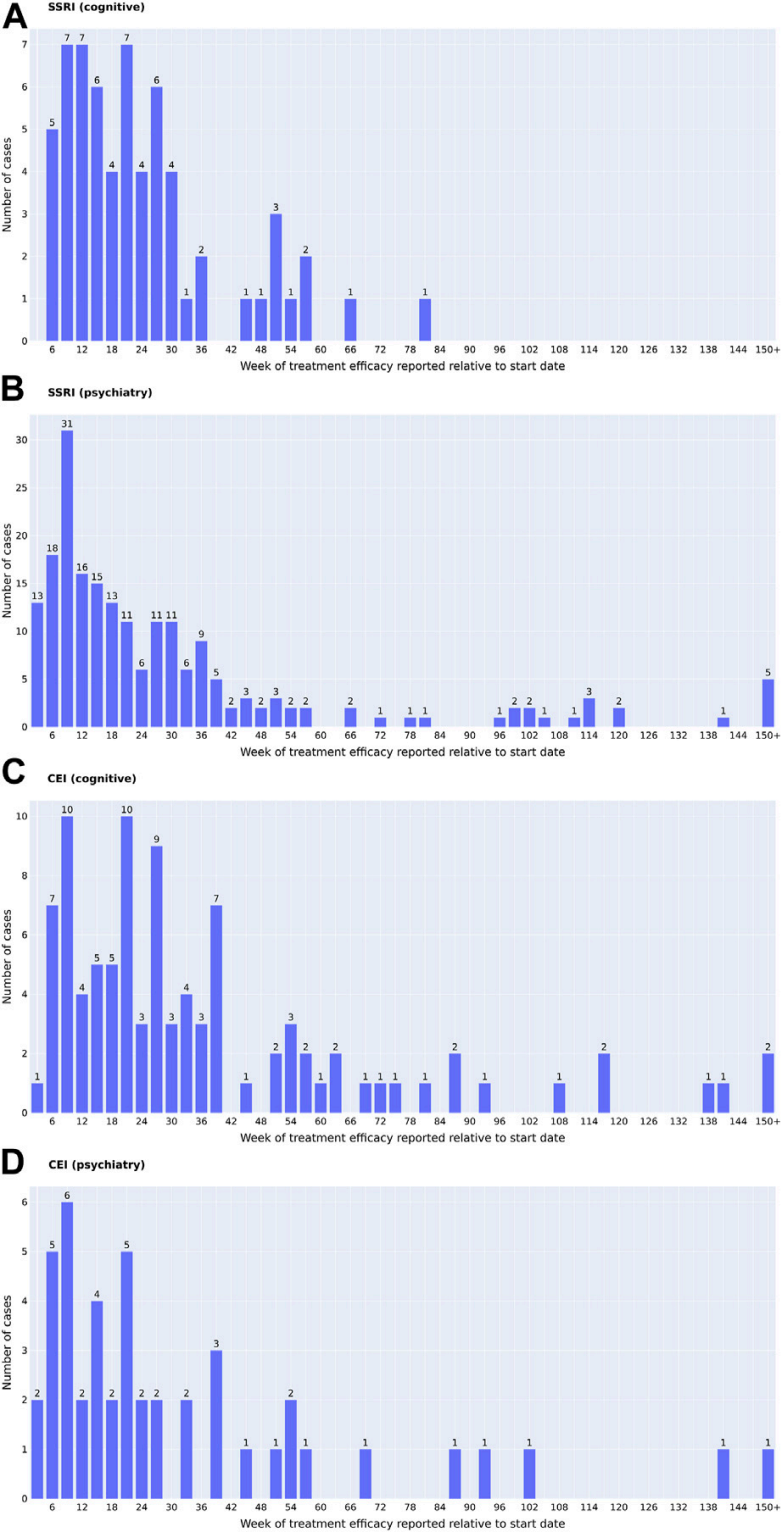
Time of the reported medication start date (**(A, B)** SSRIs; **(C, D)** CEIs) to the determination of treatment efficacy (**(A, C)** change in cognition; **(B, D)** change in psychiatric symptoms). The time of the reported intolerance cases is not included. One date of reported effect per individual is presented. For patients who had multiple dates of similar treatment effects reported, the date of reported cognitive comments that matched the dates of MMSE changes was prioritized when available. For all the psychiatric effects and the cognitive effects that did not have the matching dates of MMSE changes, the comments that were reported closest to 6 months, or 26 weeks, were prioritized.

Figure 2 characterizes cases of medication usage into patient’s age, sex, AEs, length of time of medication usage, and diagnostic codes. Similar trends in demographics were observed: 52.0% of SSRI and 47.8% of CEI cases of medication usage were women and the average ages were 73 for SSRI and 74 for CEI cases. AEs were reported in 23.6% of SSRI and 43.5% of CEI cases, while 57.3% of SSRI and 33.0% of CEI cases contained no record of tolerability or AEs during treatment. For analyzing diagnostic codes, SSRI cases had 32.4% AD, 18.7% VD, and 8.9% mixed dementia; likewise, CEI cases had 47.0% AD, 11.3% VD, and 11.3% mixed dementia (Figure 2E). The types of medications used are listed in Figure 3; the medications that were switched within the 3-month washout period for various reasons, from insurance coverage change to ineffectiveness of the prescription, are listed in the order of their usage. The most used drugs were sertraline for SSRIs and donepezil for CEIs. The time from the reported start date of medication usage to the assessment of the treatment effect is shown in Figure 4.

### Validity of the NOTE score

Inter-rater reliability tests revealed excellent inter-rater agreement (Table 2). The ICC showed 1.0 (perfect agreement) for cognitive NOTE and 0.96 (95% CI: 0.87–1.0) for psychiatric NOTE. Krippendorff’s alpha showed an alpha value of 0.90 (95% CI: 0.74–1.0) for cognitive NOTE and 0.91 (95% CI: 0.76–1.0) for psychiatric NOTE. Dice coefficients were 0.83 for “N/A,” 1.0 for “improve” and “no change,” and 0.92 for “worse” in cognitive NOTE and 1.0 for “N/A” and “improve,” 0.75 for “no change,” and 0.90 for “worse” in psychiatric NOTE.

**TABLE 2:** Inter-rater reliability results for intraclass correlation coefficients, Krippendorff’s alpha, and Dice coefficients. Values for ICCs and Krippendorff’s alpha are presented as mean (95% confidence interval) based on bootstrapping with 500 resampling iterations, using the bias-corrected and -accelerated method for estimating confidence intervals.

		Cognitive NOTE	Psychiatric NOTE
ICC (type = ICC2k) based on the ordinal assumption (worsen < no change < improved), excluding N/A values	Coefficient	1.0^a^	0.96 (0.87–1.0)
	*p*-value	<0.001	<0.001
	Number of subjects	23	24
Krippendorff’s alpha	Alpha	0.90 (0.74–1.0)	0.91 (0.76–1.0)
	Number of subjects	30	30
Dice coefficient	N/A	0.83	1.0
	Improve	1.0	1.0
	No change	1.0	0.75
	Worse	0.92	0.90

^a^ Due to the perfect agreement between raters, calculating a 95% confidence interval using bootstrapping was not feasible.

**FIGURE 5:**
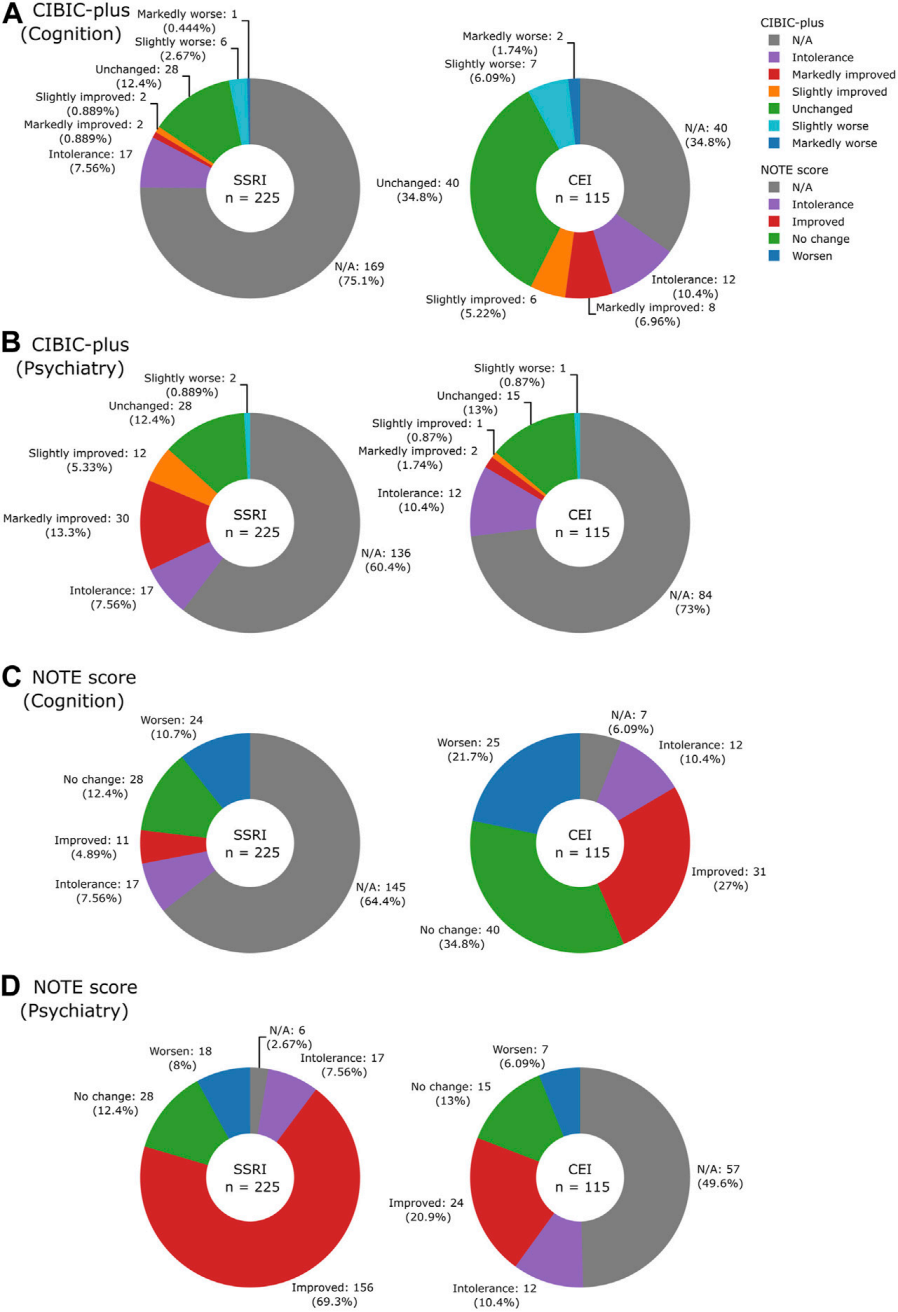
Pie charts illustrating the breakdown of CIBIC-plus: **(A)** cognition and **(B)** psychiatry and NOTE scores: **(C)** cognition and **(D)** psychiatry by drugs.

The distributions of NOTE and CIBIC-plus scores and the number of notes deemed unevaluable are shown in Figure 5. Many of the non-prioritized evaluations were missing (SSRIs 64.4% of cognitive NOTE and CEIs 49.6% of psychiatric NOTE) because they were not the target treatment of focus; the cognitive effect was not always reported after SSRI use, and the same applied to the psychiatric effect after CEI use. For CIBIC-plus, the keywords that were not readily classifiable into the seven-point scale, such as “donepezil working well,” were marked as N/A. Similarly, comments that can be classified as “moderate” were not found and therefore marked as N/A. While only 39.6% (89/225) of SSRI and 65.2% (75/115) of CEI cases could be evaluated by their prioritized CIBIC-plus, most of SSRI (219/225, 97.3%) and CEI (108/115, 93.9%) cases could be evaluated by their prioritized NOTE. The results indicated that the simplified scale of NOTE is more suitable than the detailed scale of CIBIC-plus when evaluating treatment effects from clinical notes.

**FIGURE 6:**
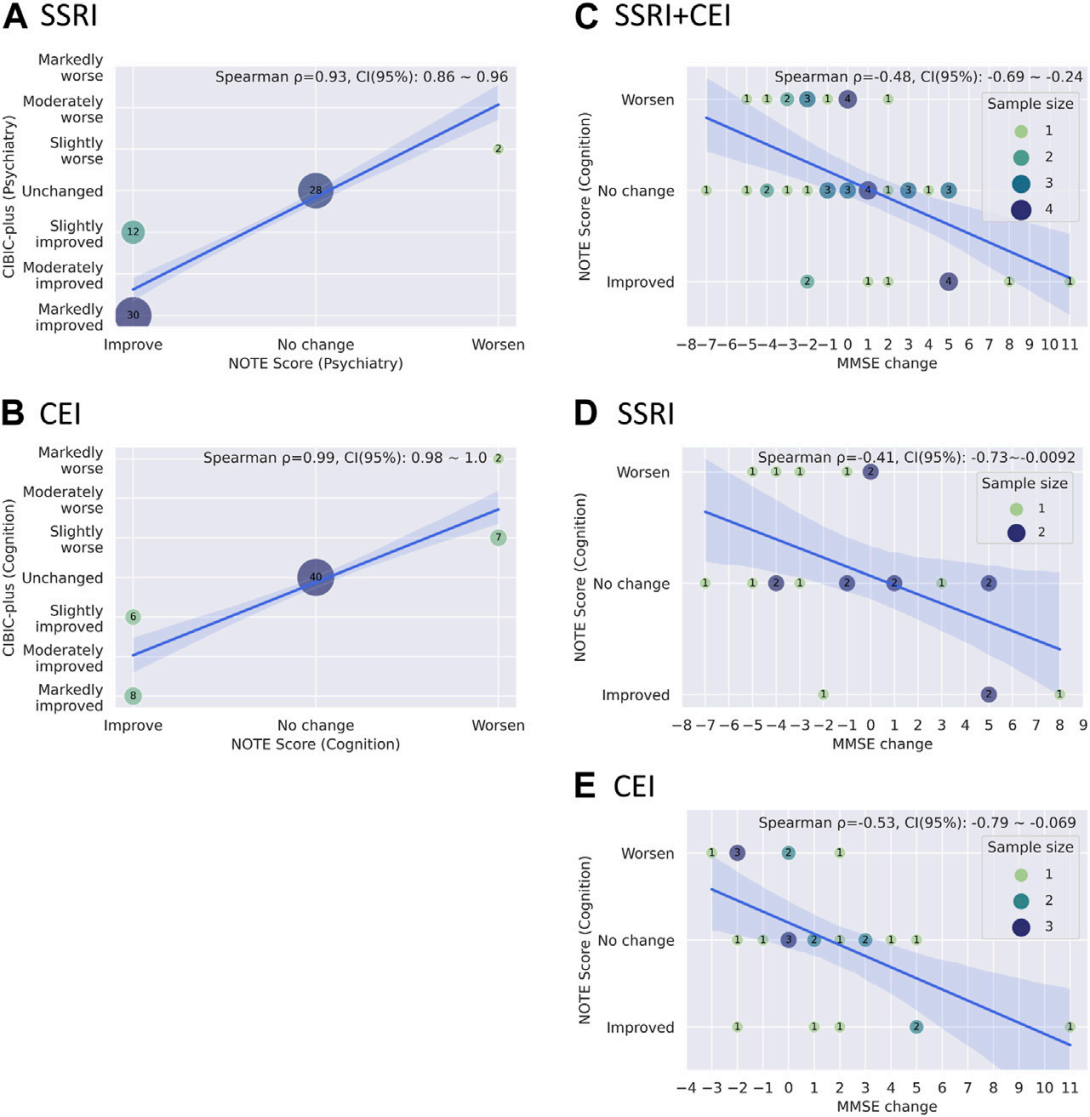
**(A, B)** Bubble plots showing the relationships between CIBIC-plus and NOTE scores through drugs **(A)** SSRI psychiatric scores and **(B)** CEI cognitive scores). **(C–E)** Bubble plots showing the relationship between MMSE changes and cognitive NOTE scores by drugs: **(C)** SSRI + CEI, **(D)** SSRI, and **(E)** CEI. A solid blue line and a light blue area represent the regression line with its 95% confidence intervals. The mean coefficient, 95% confidence interval of Spearman’s rank correlation, and the legend of sample sizes (for C–E) are shown in the upper right corner of the graph. The mean coefficient and its confidence interval are estimated based on bootstrapping with 10,000 resampling iterations, using the bias-corrected and -accelerated method.

Figures 6A and B show Spearman’s rank correlations comparing NOTE and CIBIC-plus. As expected from the simplified versus detailed nature of the two scoring systems, there were strong correlations between NOTE and CIBIC-plus for both medications: SSRIs (psychiatry) with *ρ* = 0.93 and *p* = 1.6 × 10^−31^ (bootstrap mean: 0.93, 95% CI: 0.86–0.96) and CEIs (cognition) with *ρ* = 0.99 and *p* = 2.7 × 10^−59^ (bootstrap mean: 0.99, 95% CI: 0.98–1.0). Figures 6C–E show Spearman’s rank correlations between NOTE and change in MMSE from pre- to post-medication usage. NOTE scores of SSRIs, CEIs, and both combined all correlated significantly with MMSE changes: SSRIs + CEIs (cognition) with *ρ* = −0.49 and *p* = 0.00049 (bootstrap mean: −0.48, 95% CI: −0.69 to −0.24, *n* = 47); SSRIs (cognition) with *ρ* = −0.42 and *p* = 0.0496 (bootstrap mean: −0.41, 95% CI: −0.73 to −0.0092, *n* = 22); and CEIs (cognition) with *ρ* = −0.54 and *p* = 0.0054 (bootstrap mean: −0.53, 95% CI: −0.79 to −0.069, *n* = 25).

### Treatment responses

The proportion of NOTE scores in relation to the clinical characteristics is shown in Table 3. The type II likelihood ratio test on multinomial models revealed no significant differences between diagnostic codes, sex, and age in relation to the group of responders, non-responders, and intolerances among SSRIs (diagnostic code: *p* = 0.70; sex: *p* = 0.20; age: *p* = 0.45) and CEIs (diagnostic code: *p* = 0.66; sex: *p* = 0.20; age: *p* = 0.30). Among 225 SSRI cases, 69.3% were SSRI responders, 20.4% were SSRI non-responders, and 7.6% were SSRI-intolerant. Among 115 CEI cases, 61.8% were CEI responders, 21.7% were CEI non-responders, and 10.4% were CEI-intolerant. The remaining cases (SSRI 2.7% and CEI 6.1%) did not have the prioritized psychiatric or cognitive comment available for evaluation.

**TABLE 3:** Proportion of age, sex, and diagnostic codes in relation to the results of NOTE.

Drug	Variable	Effectiveness of the NOTE score^a^						Statistics^b^
		Total	N/A^c^	Intolerance	Improved	No change	Worsen	*p*-value
SSRI	Diagnosis code, *n* (%)							0.70
	*AD^d^*	73 (100%)	3 (4.1%)	4 (5.5%)	50 (68%)	9 (12%)	7 (9.6%)	
	*VD^d^*	42 (100%)	1 (2.4%)	3 (7.1%)	27 (64%)	8 (19%)	3 (7.1%)	
	*AD + VD*	20 (100%)	1 (5.0%)	1 (5.0%)	16 (80%)	2 (10%)	0 (0%)	
	*Others*	90 (100%)	1 (1.1%)	9 (10%)	63 (70%)	9 (10%)	8 (8.9%)	
	Sex, *n* (%)							0.20
	*Woman*	117 (100%)	4 (3.4%)	11 (9.4%)	80 (68%)	10 (8.5%)	12 (10%)	
	*Man*	108 (100%)	2 (1.9%)	6 (5.6%)	76 (70%)	18 (17%)	6 (5.6%)	
	Age at the start of the medication, mean (SD)							0.45
		73 (9.7)	70 (10.4)	72 (12.7)	73 (9.2)	76 (8.6)	72 (12.4)	
	Total, *n* (%)							
		225 (100%)	6 (2.7%)	17 (7.6%)	156 (69%)	28 (12%)	18 (8.0%)	
CEI	Diagnosis code, *n* (%)							0.66
	*AD*	54 (100%)	3 (5.6%)	2 (3.7%)	15 (28%)	19 (35%)	15 (28%)	
	*VD*	13 (100%)	1 (7.7%)	2 (15%)	3 (23%)	4 (31%)	3 (23%)	
	*AD + VD*	13 (100%)	0 (0%)	1 (7.7%)	6 (46%)	4 (31%)	2 (15%)	
	*Others*	35 (100%)	3 (8.6%)	7 (20%)	7 (20%)	13 (37%)	5 (14%)	
	Sex, *n* (%)							0.20
	*Woman*	55 (100%)	6 (11%)	3 (5.5%)	13 (24%)	20 (36%)	13 (24%)	
	*Man*	60 (100%)	1 (1.7%)	9 (15%)	18 (30%)	20 (33%)	12 (20%)	
	Age at the start of the medication, mean (SD)							0.30
		74 (8.8)	79 (7.7)	77 (10.1)	73 (9.7)	74 (8.5)	73 (7.6)	
	Total, *n* (%)							
		115 (100%)	7 (6.1%)	12 (10%)	31 (27%)	40 (35%)	25 (22%)	

^a^ SSRI, psychiatric NOTE score; CEI, cognitive NOTE score.

^b^ Type II likelihood ratio test on the multinomial model.

^c^ N/A, prioritized comment on the treatment effect not available.

^d^ AD, Alzheimer’s disease; VD, vascular dementia.

## Discussion

This study aimed to find the medication responder rates of dementia patients in real-world settings. Even though our outpatient cohort included various etiologies of dementia and had no clinical exclusion criteria other than age, the response rates to CEIs and SSRIs were similar to those obtained in a study cohort with a homogeneous clinical profile targeting a specific type of dementia.

In our study, 27.0% of CEI users reported improvement and 34.8% reported stable symptoms, with a total of 61.8% CEI responders. This is consistent with what was reported in the research cohorts. According to Miranda et al. (2015), 27.8% of CEI users were good responders scoring ≥2 in MMSE and 37.1% were neutral responders scoring between −1 and +1 in MMSE at 12 months of treatment, with a total of 64.9% CEI responders. Another study reported that the donepezil responder rate in AD patients was 60% (Pilotto et al., 2009). For SSRIs, our study had 69.3% of outpatients who reported improvement. This is also consistent with what was reported in the clinical trials of agitation or depression associated with AD. According to Porsteinsson et al. (2014a), 14% of citalopram users reported marked improvement, 26% reported moderate improvement, and 29% reported minimal improvement, with a total of 69% of citalopram responders among AD patients. Likewise, Finkel et al. (2004) reported that among dementia patients with moderate-to-severe symptoms, who were on donepezil medication, 60% of sertraline users achieved a response of ≥50% reduction in a four-item neuropsychiatric inventory-behavioral subscale.

The intolerance rate was also similar to the reports based on the clinical trials. While our intolerants included 7.6% of SSRI users and 10.4% of CEI users, a systematic review of antidepressants reported that 12% of SSRI users withdrew from the trial due to AEs (Seitz et al., 2011), and another clinical trial reported that 10.7% of donepezil users and 21.8% of rivastigmine users discontinued the study due to AEs (Wilkinson et al., 2002).

Interestingly, the proportion of diagnoses (i.e., AD *vs.* VD *vs.* AD + VD *vs.* other dementias), sex, and age did not differ among intolerants, responders, and non-responders, suggesting that dementia type, sex, and age may not predict responses to CEIs or SSRIs in a heterogeneous clinical setting.

An aforementioned study by Vaci et al. (2021) investigated the effectiveness of CEIs and memantine by extracting mentions of relevant medications and MMSE/MoCA scores of dementia patients. The authors reported that 33% of them had an increase in cognitive scores and 35% had the stabilization of scores, totaling the treatment responder rates to 68% of individuals. While our responder rates are similar, the main difference between the study by Vaci et al. (2021) and our EMR study is that in addition to cognitive performances, we focused on the overall treatment effects of SSRIs and CEIs addressing problematic psychiatric and cognitive symptoms. For example, if patients and caregivers feel that the medications are helping by keeping them more motivated and interactive or by having fewer nighttime disruptions and agitations, those are essential treatment effects that directly affect the quality of life of patients and caregivers; NOTEs take that into account and quantifies them.

A unique quality of this study is its utilization of clinical comments in EMRs and analysis of natural language to assess pharmacotherapeutic responses using a simple Likert scale. Clinical trials investigating the treatment effects of dementia drugs have widely used the CIBIC-plus assessment in the past (Stanley et al., 2021). CIBIC-plus has the advantage of detailing the degree of change by having a clinician interview patients and caregivers (Sheehan, 2012; Stanley et al., 2021). However, the degree of detail necessary to translate a clinical note into a CIBIC-plus score is rarely available. Thus, in studies that involve retrospective analysis of EMRs without additional patient contact, NOTE provides a more flexible and effective measure.

Sophisticated scales have been used in trials to assess the cognitive and functional status of dementia patients, including the Alzheimer’s Disease Assessment Scale–Cognitive Subscale (Kueper et al., 2018) and the Bristol Activities of Daily Living Scale (Sheehan, 2012); nonetheless, these are not routinely used in clinical practice. Although the Cornell Scale and the Neuropsychiatric Inventory Questionnaire are used in clinical practice to assess neuropsychiatric symptoms, these scales are not always systematically obtained and recorded in EMRs, making it impractical to analyze neuropsychiatric changes after medication use.

## Limitations

This study includes several limitations. First, the manual review of EMRs was labor-intensive in identifying and interpreting the information of interest, which limited the study sample size. Due to the limited sample size, the potential confounding effects of various comorbidities, commonly experienced by this age group, and potential drug interactions between non-dementia-related medications were not analyzed. Furthermore, because this was a retrospective observation of available medical records, we were unable to predefine the observation period, and therefore, the dates of clinical comments on the treatment effect were found to be varied widely. Moreover, with studies that involve human reviewers’ interpretations of documents, there are concerns for subjectivity and human error (Wang et al., 2019), despite NOTE demonstrating excellent inter-rater reliability. We expect these limitations can be addressed by incorporating artificial intelligence. The manual review presented in this study can be an important first step that can be utilized to develop a reference standard used to build the natural language processing (NLP) model for structuring clinical data of dementia patients for further automated, large-scale analyses of EMRs (Vaci et al., 2020; Sheu et al., 2021; Vaci et al., 2021). In the same light, we chose to focus on CEIs and SSRIs in this study because these two classes of medications are the most commonly used in our study cohort. Once the large-scale analyses of EMRs become possible with NLP to substantially increase the study sample size, we would be able to extend our research on medications such as memantine and SNRIs in future studies and analyze the confounding effects of comorbidities and potential drug interactions. Second, our analysis was performed on EMRs in American English. Clinical text may naturally and structurally differ between healthcare systems, so the English words found and categorized in our study may not be fully generalizable to EMRs of other healthcare systems; adjustments may be necessary if similar analyses were to be conducted at other sites. Third, the observed response rates are a product of who is prescribed medication. For example, if clinicians are more likely to prescribe medications to patients they believe to be high responders, then response rates may be higher than those observed if treatments were randomly assigned to a heterogeneous population. Finally, there are no standardized data on the overall response to medications. Even though significant correlations were observed between cognitive NOTEs and MMSE changes for both medications, NOTEs were derived from clinicians’ impressions or patient and caregiver reports on how they felt about the medication effects, which can involve subjectivity and bias on its own. Despite these limitations, it remains critical to know how effective a medication is and what those experiences are in the population receiving that medication.

## Conclusion

Responses to CEIs and SSRIs prescribed to outpatients were similar to those previously reported in clinical trials. Based on our results, medication responses are difficult to predict from the type of dementia, sex, and age of a patient. The three-point Likert scale NOTE introduced in this study is suitable for extracting overall treatment responses to drugs recorded in EMRs and has the potential to be used to train NLP models for large-scale automated analyses in the future.

## Data Availability

The data analyzed in this study are subject to the following licenses/restrictions: to protect patient healthcare information, the raw electronic medical record data used in this study cannot be shared. HIPAA defined that limited or fully de-identified data will be shared with researchers who have an appropriate IRB approval, have been qualified through the Johns Hopkins Richman Family Precision Medicine Center of Excellence in Alzheimer’s Disease Data Sharing Committee, and have signed a data use agreement with Johns Hopkins. Requests to access these datasets should be directed to the Johns Hopkins Richman Family Precision Medicine Center of Excellence in Alzheimer’s Disease Data Sharing Committee, https://www.hopkinsmedicine.org/inhealth/precision-medicine-centers/alzheimers/.
